# Evaluation of the concordance between nasopharyngeal and oropharyngeal swabs in the detection of COVID-19

**DOI:** 10.4102/jphia.v17i1.1236

**Published:** 2026-02-26

**Authors:** Michel C. Tommo Tchouaket, Joseph Fokam, Ezéchiel N. Semengue, Yagai Bouba, Collins A. Chenwi, Alex D. Nka, Mirice Mbazo’o, Désiré Takou, Samuel M. Sosso, Grâce A. Beloumou, Aude C. Ka’e, Aurelie M. Ngueko, Nadine Fainguem, Laeticia Y. Heunko, Sandrine C. Ndjeyep, Willy L. Pabo, Davy-Hyacinthe G. Anguechia, Naomi-Karell Etame, Evariste Molimbou, Rachel A. Mundo, Aissatou Abba, Gregory-Edie Halle-Ekane, Anne-Cecile Z.-K. Bissek, Vittorio Colizzi, Carlo-Federico Perno, Alexis Ndjolo

**Affiliations:** 1 Chantal Biya International Reference Centre for Research on HIV/AIDS Prevention and Management (CIRCB), Yaoundé, Cameroon; 2 School of Health Sciences, Catholic University of Central Africa, Yaoundé, Cameroon; 3 Faculty of Health Sciences, University of Buea, Buea, Cameroon; 4 Faculty of Science and Technology, Evangelical University of Cameroon, Bandjoun, Cameroon; 5 University of Rome ‘Tor Vergata’, Rome, Italy; 6 Faculty of Medicine and Biomedical Sciences, University of Yaoundé I, Yaoundé, Cameroon; 7 Faculty of Science and Technology, University of Yaoundé I, Yaoundé, Cameroon; 8 School of Health Sciences, Catholic University of Central Africa, Yaoundé, Cameroon; 9 Division of Health Operational Research, Ministry of Public Health, Yaoundé, Cameroon; 10 Bambino Gesu Pediatric Hospital, Rome, Italy

**Keywords:** concordance, COVID-19, nasopharyngeal swabs, oropharyngeal swabs, detection

## Abstract

**Background:**

Nasopharyngeal swabs (NASO) cause discomfort for patients, which can discourage them from getting tested for COVID-19 and limit case detection. It is therefore necessary to consider an alternative, more comfortable swab.

**Aim:**

Evaluated the concordance between nasopharyngeal and oropharyngeal sampling for COVID-19 diagnosis in the Cameroonian context.

**Setting:**

This study was carried out at “Chantal Biya” International Reference Centre for Research on HIV/AIDS Prevention and Management (CIRCB) in Yaoundé, Cameroon.

**Methods:**

A comparative study was conducted in April 2021 among consenting participants tested for COVID-19 at “Chantal Biya” International Reference Centre for Research on HIV/AIDS Prevention and Management (CIRCB) in Yaoundé, Cameroon. Sampling began with nasopharyngeal swabs, followed by oropharyngeal swabs, all taken by the same technician, and analysis was carried out using polymerase chain reaction (PCR) tests on the Abbott platform. Statistical analyses were performed using GraphPad version 6.0; *p* < 0.05 was considered statistically significant.

**Results:**

A total of 154 participants were enrolled (59.7% male, median age 38 years, interquartile range [IQR]: 30–49). Following PCR testing, the overall COVID-19 positivity rate was 36.36% (56/154), with 34.41% (*n* = 53/154) in nasopharyngeal versus 16.23% (*n* = 25/154) in oropharyngeal samples, *p* < 0.0002. The overall concordance rate was 78% (*n* = 120/154), with 39.28% positive concordance and 74.24% negative concordance (kappa = 0.441 [0.289–0.513]). According to severe acute respiratory syndrome coronavirus 2 viral load, the positive concordance was improved with high viral load (cycle threshold [CT]: ≤ 25): 61% (*n* = 11/18) versus 31% (*n* = 11/35) with low viral load (CT > 25), *p* = 0.037; odds ratio (OR) = 3.43. According to gender, the positive concordance was higher in men, 55% (*n* = 16/29), versus 25% (*n* = 6/24) in women, *p* = 0.021; OR = 0.27. Using nasopharyngeal swab as the gold standard, oropharyngeal swab had a sensitivity of 41.50% (*n* = 22/53), specificity 97.02% (*n* = 98/101), positive predictive value (PPV) 88% (*n* = 22/25) and negative predictive value (NPV) 76% (*n* = 98/129).

**Conclusion:**

Our evidence suggests a superiority effect of nasopharyngeal in detecting cases of COVID-19. However, the overall high PPV of oropharyngeal swab, and its improved performance with high viral load.

**Contribution:**

Therefore, in case of counter-indication to nasopharyngeal swabbing, oropharyngeal can be an acceptable alternative.

## Introduction

The new severe acute respiratory syndrome coronavirus 2 (SARS-CoV-2), which belongs to the Coronaviridae family of viruses, is thought to have originated in or around Wuhan, China.^1,2^ The most widespread theory of origin describes virulence acquired through a zoonotic event. Rapid transmission, asymptomatic but transmissible infection, poor detection capability, ineffective treatment methods and the absence of early contact and quarantine measures have led to unprecedented global spread.^3,4^ The ability of the SARS-CoV-2 virus to spread rapidly through the global population via respiratory droplets, often in the absence of symptoms, is remarkable. Affecting almost every country and demographic group, COVID-19 has rapidly overwhelmed many of the world’s hospitals and much of the healthcare infrastructure.^5,6^ In March 2020, the World Health Organization (WHO) declared COVID-19 a public health event of international concern. By August 2021, SARS-CoV-2 had infected around 200 million people and killed more than 4.2 million worldwide, making it one of the deadliest epidemics in human history.^7^ Faced with this unprecedented challenge, countries implemented guidelines aimed at limiting human-to-human spread, including widespread quarantine/isolation, compulsory wearing of masks and extensive home-keeping instructions, involving the closure of gatherings and businesses, and finally, systematic screening of symptomatic people and contact cases.^1,8^

Screening for SARS-CoV-2 was preferably performed using nasopharyngeal swab (NPS) sampling with flocked swabs. Nasopharyngeal swabbing is a pharyngeal mucus sampling technique used in real-time polymerase chain reaction (RT-PCR) tests for COVID-19 (reference test).^9^ The swab is inserted through the nostril into the nasopharynx and as many cells as possible are collected by gently rotating the swab. This sampling method is considered to have the highest diagnostic yield, as evidenced by its use as a reference method by the United States (US) Food and Drug Administration (FDA).^1,10^ However, nasopharyngeal sampling requires professional sampling (a major limitation given the constraints of the healthcare system in almost all parts of the world) and protective equipment that is generally inadequate, as well as the flocked swabs themselves.^3^ In addition, NPS collection is uncomfortable, limiting patients’ willingness to come forward for testing, particularly if they are asymptomatic or, in some cases, have mild symptoms. Collection difficulties can compromise case detection and, consequently, have an impact on epidemiological surveillance.^5,7^ These problems have led to a decrease in the number of patients for virological surveillance since the start of the COVID-19 pandemic.^11^ It therefore seems crucial to propose an alternative method to nasopharyngeal swabbing to optimise screening of negative and positive patients, in order to improve virological monitoring and control the pandemic.^8^

Since the start of the COVID-19 pandemic, medical staff have encountered difficulties in carrying out virological surveillance of suspected COVID-19 patients using NPS, because of the following two recommendations in France: a systematic SARS-CoV-2 virological test (NPS) must be carried out in specific diagnostic centres and the obligation to receive the result very quickly.^12,13^ As a result, Sentinel doctors and patients are reluctant to take NPSs for virological surveillance at a COVID-19 diagnostic centre. These problems have led to a decline in the number of patients included in virological surveillance of patients since the start of the COVID-19 pandemic. In view of the above, offering an alternative method to nasopharyngeal swabbing is therefore a necessity in order to control the pandemic.^14,15^ Diagnosis of COVID-19 is based on analysis of a naso-oropharyngeal swab by PCR. As COVID-19 is still a pandemic, the use of this diagnostic test is likely to remain valid for some time. A patient with symptoms of COVID-19 presented with unilateral cerebrospinal fluid rhinorrhoea after nasopharyngeal swabbing. Although NPSs are very common, the complication rate is very low. However, life-threatening complications may occur in rare cases, and caution is advised.

Oropharyngeal swabs (OPS) are seen as a second method for diagnosing the disease and in order to better understand the effectiveness of this type of sampling in field situations, such as the surveillance of circulating respiratory viruses. Thus, the aim of this study was to evaluate the concordance between nasopharyngeal and oropharyngeal sampling for COVID-19 diagnosis in the Cameroonian context.

## Research methods and design

### Study design and participants

As part of the implementation of the project European and Developing Countries Clinical Trial Partnership (EDCTP) performance evaluation of COVID-19 tests (PERFECT)-Study in Cameroon whose main objective was (to accelerate the development of new or improved medical interventions for poverty-related infectious diseases – human immunodeficiency virus (HIV), tuberculosis (TB), malaria, neglected infectious diseases, diarrhoeal diseases, lower respiratory tract infections and emerging and re-emerging infectious diseases), a cross-section and comparative study was conducted in April 2021 among all participants tested for COVID-19 at “Chantal BIYA” International Reference Centre for Research on HIV/AIDS Prevention and Management (CIRCB) in Yaoundé, Cameroon.

### Method and study population

**FIGURE 1:**
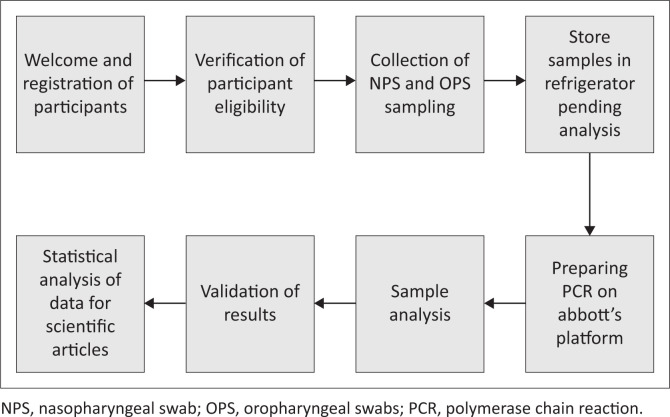
Flow chart of participant recruitment and sample processing for greater clarity.

A standard questionnaire was administered to participants by investigators trained in the study protocol, covering clinical and socio-demographic characteristics. Participants were consecutively enrolled based on the following inclusion criteria: (1) all participants presenting for COVID-19 testing at the CIRCB during the month of April 2021 and (2) all persons who gave their consent to participate in the study (see Figure 1).

### Data collection

All clinical specimens in this study were taken from patients who came to the CIRCB for the diagnosis of COVID-19. Generally speaking, swabs were taken by qualified personnel in a 1-mL tube containing viral transport medium (VTM), in accordance with the manufacturer’s instructions and in compliance with universal biosafety measures. In all project participants, sampling began with nasopharyngeal sampling, followed by oropharyngeal sampling, all performed by the same technician. After sampling, the samples were stored at 4 °C to 8 °C for 2 h to 5 h, while the analyses were prepared in the laboratory. All PCR analyses were performed on the Abbott platform.

Nucleic acid extraction, amplification and detection with Abbott platform: For Abbott rRT-PCR assay for SAR-CoV-2 detection, a 1000-µL aliquot of each inactivated sample (500 µL NPS + 500 µL of deoxyribonuclease-ribonuclease [DNase-RNase] free water heated at 70 °C for 10 min) was loaded into Abbott m2000sp instrument, combined with the Abbott SARS-CoV-2 master mix containing an internal ribonucleic acid (RNA) control, primers, and probes targeting both an RNA-dependent RNA polymerase (*RdRp*) gene, specific for SARS-CoV-2 as well as the conserved structural protein nucleocapsid (*N*) gene (www.fda.gov/media/136258/download).

Amplification was performed using the thermocycler m2000rt after automated extraction and sample preparation using the Abbott m2000sp instrument. At the end of the amplification process, negative results were rendered as ‘target not detected’ (no amplification was observed after 37 cycle numbers); meanwhile, positive results were rendered as ‘target detected’ with a given number representing the cycle number (CN) at which the detection phase was initiated. This CN value was inversely proportional to the viral load of the patient. According to the manufacturer, the detection sensitivity for this assay is 100 copies/mL.

### Description of the study site

“Chantal BIYA” International Reference Centre for Research on HIV/AIDS Prevention and Management is a national reference laboratory for the molecular diagnosis of COVID-19 under the Ministry of Public Health of Cameroon. To ensure reliability in COVID-19 testing, CIRCB participates in external quality control programmes for proficiency testing with the WHO and the African Society of Laboratory Medicine (ASLM). A total of 41 716 samples have been tested so far. “Chantal BIYA” International Reference Centre for Research on HIV/AIDS Prevention and Management also has a sequencing platform dedicated to human immunodeficiency virus (HIV) genotyping and activities related to the COVID-19 genomic surveillance platform in Cameroon.

### Clinical and laboratory procedures

#### Biological sampling methods

Polymerase chain reaction tubes containing VTM and a swab (VWR part number 300260) were used for sample collection. Consecutive NPS and OPS samples were taken in parallel and inserted into the VTM tubes. The NPS samples were always taken first. These were obtained by gently pushing the swab deep into the nostril (up to the nasopharynx) and scraping off as many cells as possible from the inner surface of the nostril. A few seconds were observed before sampling the second tube: OPS. The OPS samples were obtained by rubbing the posterior wall of the oropharynx and rotating them once in each direction. They were then transferred to the tube by rubbing the posterior wall of the oropharynx and rotating them once in each direction. They were then transferred to the tube. The various NPS and OPS samples were collected in accordance with Ottawa Public Health recommendations (https://www.ottawapublichealth.ca/en/professionals-and-partners/how-to-collect-a-nasopharyngeal--np--swab.aspx). The collected samples were analysed using the Abbott platform, according to the manufacturer’s instructions (https://www.molecular.abbott/int/en/products/infectious-disease/RealTime-SARS-CoV-2-Assay), targeting the *RdRp* gene and the *N* gene.

Samples, where no transport medium was available, were considered inadequate.

### Results interpretation

Positive for SARS-CoV-2: Internal control < 33 cycle threshold (CT) and *RdRp* and *N* gene were < 37 CT (for Abbott). Negative for SARS-CoV-2: Internal control < 33 CT and *RdRp* and *N* gene were ≥ 37 CT (for Abbott); all CT ≤ 25 was high viral load and CT > 25 was low viral load.

### Statistical analysis

Data were entered into an Excel spreadsheet and statistical analyses were performed using GraphPad version 6 software; correlation analyses were performed using Spearman’s correlation test; concordance in diagnosis was assessed according to Cohen’s kappa value (*k*), and results were interpreted according to the criteria proposed by Landis & Koch: *k* = 0.01–0.20 (low agreement), *k* = 0.21–0.40 (medium agreement), *k* = 0.41–0.60 (moderate agreement), *k* = 0.61–0.80 (high agreement) and *k* = 0.81–1.00 (almost perfect agreement). All *p*-values < 0.05 were considered statistically significant, with a 95% confidence interval.

### Ethical considerations

Ethical clearance to conduct this study was obtained from the National Human Health Research Ethics Committee (CNERSH) (No. 2022/01/1430/CE/CNERSH/SP). Per the Helsinki declaration and the national regulations, informed consent was obtained from all participants; confidentiality was ensured through de-identification by the use of a unique identifier for each participant and the storage of data in a password-protected computer.

## Results

### Socio-demographic characteristics of the study population

A total of 154 participants were recruited, 59.74% (*n* = 92/154) male and 40.25% (*n* = 62/154) female. The median age of the participants was 38 years (interquartile range [IQR]: 30–49), with the most represented age group being those aged between 16 years and 45 years (59.74%).

Overall, the COVID-19 positivity rate was 36.36% (*n* = 56/154), with 34.41% (*n* = 53/154) in nasopharyngeal samples versus 16.23% (*n* = 25/154) in oropharyngeal samples, *p* < 0.0002.

By gender, the positivity rate was moderate in male participants, 57.14% (*n* = 32/56), than in female participants, 42.85% (*n* = 24/56), *p* = 0.61.

According to SARS-CoV-2 viraemia, positive concordance was higher with high viral load (CT ≤ 25): 61% (*n* = 11/18) versus 31% (*n* = 11/35) low viral load (CT > 25), *p* = 0.037; odds ratio (OR) = 3.43. By gender, positive concordance was higher in men, 55% (*n* = 16/29) versus 25% (*n* = 6/24) in women, *p* = 0.021; OR = 0.27.

For clinical symptoms, the positive agreement was 40% (*n* = 2/5) for symptomatic participants versus 42% (*n* = 20/48) for asymptomatic participants, *p* = 0.94.

### COVID-19 polymerase chain reaction positivity

According to Table 1, more men, 57% (*n* = 32/56), were infected than women, 43% (*n* = 24/56), without a significant association.

**TABLE 1:** Baseline viral load of severe acute respiratory syndrome coronavirus 2 according to gender and age.

Variable	Positive	Negative	Total	*p*-value
**Baseline viral load of SARS-CoV-2 according to Gender**				0.6100
Female	24	38	62	-
Male	32	60	92	-
Total	56	98	154	-
**Baseline viral load of SARS-CoV-2 according to age (years)**				
0–15	2	10	12	0.1600
16–45	34	58	92	< 0.0001
≤ 45	20	30	50	-
Total	56	98	154	-

SARS-CoV-2, severe acute respiratory syndrome coronavirus 2.

There was a statistically significant association between SARS-CoV-2 and age and gender distribution.

### Concordance between tests and factors influencing the level of concordance

By gender, the positive concordance was higher in men, 55% (*n* = 16/29) than 25% (*n* = 6/24) in women, *p* = 0.021; OR = 0.27.

For clinical symptoms, the positive concordance was 40% (*n* = 2/5) for symptomatic participants versus 42% (*n* = 20/48) for asymptomatic participants, *p* = 0.94.

Using the NPS as the gold standard, the sensitivity of the OPS test was 41.50% (*n* = 22/53), specificity 97.02% (*n* = 98/101); positive predictive value (PPV) 88% (*n* = 22/25) and NPV 76% (*n* = 98/129).

As per Table 2 the overall concordance rate was 78%, with 39.28% positive concordance and 74.24% negative concordance.

**TABLE 2:** Concordance between the two types of sampling.

Oropharyngeal	Nasopharyngeal		
	Positive	Negative	Total
Positive	22	3	25
Negative	31	98	129
**Total**	**53**	**101**	**154**

## Discussion

The objective of this study was to assess the concordance between NPS and OPS swabs for the diagnosis of COVID-19 in the Cameroonian context. With the increased need for testing worldwide, accurate and easy-to-use collection methods that can assist in patient surveillance and monitoring are required. According to gender in this study, it can be generally said that men were more represented than women although this did not reveal any statistically significant association. Our study also shows that the male population was slightly higher than the female population. This difference corroborates with some studies, such as the one conducted in Wuhan, where it was found that men are more likely to have COVID-19 and to develop severe forms, which is because of their high angiotensin-converting enzyme-2 (ACE2) receptor level.^16,17^ Initially, the ACE2 encoded by the *ACE 2* gene was found to be the receptor for SARS coronavirus (SARS-CoV) and human respiratory coronavirus.^18^ Several studies conducted in China and Europe have shown that the expression of these genes in human lungs is higher in Asian men than in women.^19^ These studies quantified the expression of these genes in human cells as a function of ethnicity.^20,21^ These studies quantified the expression of ACE-2 proteins in human cells as a function of gender and ethnicity.^22^ It was shown that circulating levels of these genes are higher in men than in women and in patients with diabetes or cardiovascular disease, so men are more susceptible to disease than women.^23,24,25^ This study reported that there was a statistically significant association between male sex and COVID-19 positivity and that people over 45 years of age were more likely to have COVID-19, and several studies have reported that older patients are more likely to develop severe forms of the disease.^23,26^

Our evidence showed that there was a strong concordance between the two types of samples, with this association being statistically significant, especially in the presence of high viral load, which was not the case for low viral load.^27^ The work of Wang et al.^28^ in China reported that the detection rate of SARS-CoV-2 was higher in nasal swabs (63% [*n* = 5/8]) than in pharyngeal swabs (32% [126/398]), while another small sample study analysed 17 patients in the early stages of COVID-19 and found that a higher viral load was detected in the nose than in the throat. Nasopharyngeal swabbing remains the most effective type of swab for disease surveillance; however, in symptomatic individuals (in whom viral load was also elevated after testing), results were concordant for both NPS and OPS swabs, suggesting that in symptomatic individuals, oropharyngeal swabbing may be an option for disease diagnosis.^16,28,29^

Overall, the positivity rate was 36.36% (*n* = 56/154), with 34.41% (*n* = 53/154) in nasopharyngeal samples versus 16.23% (*n* = 25/154) in oropharyngeal samples, *p* < 0.0002. These results obtained in this study show a strong discordance between NPS and OPS samples with regard to the detection of SARS-CoV-2 RNA; these results are similar to several studies performed worldwide,^30,31^ and although NPS sampling is considered by most patients as too unpleasant, it should be noted that it is the reference specimen for COVID-19 worldwide, and according to several authors, OPS specimens are of inferior quality; OPS specimens appear to have lower viral RNA loads than NPS specimens.^32,33^

Limitations of our study include the relatively small sample size, and further evaluation would be required to reach a definitive conclusion. We could not collect enough data from most of the participants. A subsequent study will be carried out to assess the concordance of NPS versus OPS samples with antigenic tests. The statistical analyses were also underpowered and should be interpreted with caution.

## Conclusion

These results suggest that, although OPSs are not a perfect alternative to NPSs for SARS-CoV-2, their performance becomes more effective in cases of high-level (super-propagating) viraemia. Thus, OPSs could be offered to patients with a contraindication to nasopharyngeal swabbing. Nevertheless, the increased versatility of testing offered by these surrogates should be welcomed in the context of the global COVID-19 pandemic. All this knowledge about the potential comparison between OPS and NPS swabs may also be useful for other diseases.

## References

[1] Pal M, Berhanu G, Desalegn C, Kandi V. Severe acute respiratory syndrome coronavirus-2 (SARS-CoV-2): An update. Cureus. 2023;12(3):e7423. 10.7759/cureus.7423 PMC7182166 32337143

[2] Ullah R, Rana MS, Qadir M, Usman M, Ahmed N. Coronavirus pandemic: A major public health crisis for the developed and developing world. J Infect Dev Ctries. 2021;15(03):366–369. 10.3855/jidc.12995 33839711

[3] Souty C, Masse S, Valette M, et al. Baseline characteristics and clinical symptoms related to respiratory viruses identified among patients presenting with influenza-like illness in primary care. Clin Microbiol Infect. 2019;25(9):1147–1153. 10.1016/j.cmi.2019.01.014 30703528 PMC7172742

[4] Konda M, Dodda B, Konala VM, Naramala S, Adapa S. Potential zoonotic origins of SARS-CoV-2 and insights for preventing future pandemics through one health approach. Cureus. 2020;12(6):e8932. 32760632 10.7759/cureus.8932 PMC7392364

[5] Hidalgo J, Rodríguez-Vega G, Pérez-Fernández J, editors. The sudden appearance of SARS-CoV-2. In: COVID-19 pandemic. Amsterdam: Elsevier, 2022; p. 1–21.

[6] Mallah SI, Ghorab OK, Al-Salmi S, et al. COVID-19: Breaking down a global health crisis. Ann Clin Microbiol Antimicrob. 2021;20:35. 10.1186/s12941-021-00438-7 34006330 PMC8129964

[7] Cucinotta D, Vanelli M. WHO declares COVID-19 a pandemic. Acta Biomed. 2020;91(1):157–160. 32191675 10.23750/abm.v91i1.9397 PMC7569573

[8] Filip R, Gheorghita Puscaselu R, Anchidin-Norocel L, Dimian M, Savage WK. Global challenges to public health care systems during the COVID-19 pandemic: A review of pandemic measures and problems. J Pers Med. 2022;12(8): 1295. 10.3390/jpm12081295 36013244 PMC9409667

[9] Péré H, Podglajen I, Wack M, et al. Nasal swab sampling for SARS-CoV-2: A convenient alternative in times of nasopharyngeal swab shortage. J Clin Microbiol. 2020;58(6):e00721–20. 10.1128/JCM.00721-20 32295896 PMC7269411

[10] Manoj A, Bhuyan M, Raj Banik S, Ravi Sankar M. 3D printing of nasopharyngeal swabs for COVID-19 diagnose: Past and current trends. Mater Today Proc. 2021;44:1361–1368. 10.1016/j.matpr.2020.11.505 33262931 PMC7687488

[11] Sanz-Muñoz I, Castrodeza Sanz J, Eiros JM. Surveillance of COVID-19 after the pandemic. How do we do it? Med Clin Engl Ed. 2022;159(8):396–400. 10.1016/j.medcle.2022.05.017 PMC9547176 36247070

[12] Landoas A, Cazzorla F, Gallouche M, et al. SARS-CoV-2 nosocomial infection acquired in a French university hospital during the 1st wave of the Covid-19 pandemic, a prospective study. Antimicrob Resist Infect Control. 2021;10:114. 10.1186/s13756-021-00984-x 34353356 PMC8339707

[13] Alvarez E, Bielska IA, Hopkins S, et al. Limitations of COVID-19 testing and case data for evidence-informed health policy and practice. Health Res Policy Syst. 2023;21(1):11. 36698202 10.1186/s12961-023-00963-1 PMC9876649

[14] Clerici B, Muscatello A, Bai F, et al. Sensitivity of SARS-CoV-2 detection with nasopharyngeal swabs. Front Public Health. 2021;8:593491. 10.3389/fpubh.2020.593491 33575241 PMC7870983

[15] Hitzenbichler F, Bauernfeind S, Salzberger B, Schmidt B, Wenzel JJ. Comparison of throat washings, nasopharyngeal swabs and oropharyngeal swabs for detection of SARS-CoV-2. Viruses. 2021;13(4):653. 10.3390/v13040653 33920072 PMC8069237

[16] Datta PK, Liu F, Fischer T, Rappaport J, Qin X. SARS-CoV-2 pandemic and research gaps: Understanding SARS-CoV-2 interaction with the ACE2 receptor and implications for therapy. Theranostics. 2020;10(16):7448–7464. 10.7150/thno.48076 32642005 PMC7330865

[17] Young S, Taylor SN, Cammarata CL, et al. Clinical evaluation of BD veritor SARS-CoV-2 point-of-care test performance compared to PCR-based testing and versus the Sofia 2 SARS antigen point-of-care test. J Clin Microbiol. 2020;59(1):e02338–20. 10.1128/JCM.02338-20 33023911 PMC7771450

[18] Fawzy MS, Ashour H, Shafie AAA, et al. The role of angiotensin-converting enzyme 2 (ACE2) genetic variations in COVID-19 infection: A literature review. Egypt J Med Hum Genet. 2022;23(1):97. 10.1186/s43042-022-00309-6 37521836 PMC9142348

[19] Li Q, Guan X, Wu P, et al. Early transmission dynamics in Wuhan, China, of novel coronavirus-infected pneumonia. N Engl J Med. 2020;382(13):1199–1207. 31995857 10.1056/NEJMoa2001316 PMC7121484

[20] Qi S, Ngwa C, Morales Scheihing DA, et al. Sex differences in the immune response to acute COVID-19 respiratory tract infection. Biol Sex Differ. 2021;12(1):66. 10.1186/s13293-021-00410-2 34930441 PMC8686792

[21] Maltezou HC, Tseroni M, Vorou R, et al. Preparing dental schools to refunction safely during the COVID-19 pandemic: An infection prevention and control perspective. J Infect Dev Ctries. 2021;15(01):22–31. 10.3855/jidc.14336 33571142

[22] Kaseb AO, Mohamed YI, Malek AE, et al. The impact of angiotensin-converting enzyme 2 (ACE2) expression on the incidence and severity of COVID-19 infection. Pathogens. 2021;10(3):379. 33809851 10.3390/pathogens10030379 PMC8004186

[23] Ciarambino T, Para O, Giordano M. Immune system and COVID-19 by sex differences and age. Womens Health. 2021;17:17455065211022262. 10.1177/17455065211022262 PMC8188967 34096383

[24] Bwire GM. Coronavirus: Why men are more vulnerable to Covid-19 than women? Sn Compr Clin Med. 2020;2(7):874–876. 32838138 10.1007/s42399-020-00341-w PMC7271824

[25] Jakhmola S, Baral B, Jha HC. A comparative analysis of COVID-19 outbreak on age groups and both the sexes of population from India and other countries. J Infect Dev Ctries. 2021;15(03):333–341. 10.3855/jidc.13698 33839706

[26] Getachew B, Tizabi Y. Vitamin D and COVID-19: Role of ACE2, age, gender, and ethnicity. J Med Virol. 2021;93(9):5285–5294. 33990955 10.1002/jmv.27075 PMC8242434

[27] Gemmati D, Bramanti B, Serino ML, Secchiero P, Zauli G, Tisato V. COVID-19 and individual genetic susceptibility/receptivity: Role of ACE1/ACE2 genes, immunity, inflammation and coagulation. might the double X-chromosome in females be protective against SARS-CoV-2 compared to the single X-chromosome in males? Int J Mol Sci. 2020;21(10): 3474. 10.3390/ijms21103474 32423094 PMC7278991

[28] Wang W, Xu Y, Gao R, et al. Detection of SARS-CoV-2 in different types of clinical specimens. JAMA. 2020;323(18):1843–1844. 32159775 10.1001/jama.2020.3786 PMC7066521

[29] Lan J, Ge J, Yu J, et al. Structure of the SARS-CoV-2 spike receptor-binding domain bound to the ACE2 receptor. Nature. 2020;581(7807):215–220. 10.1038/s41586-020-2180-5 32225176

[30] Todsen T, Tolsgaard M, Folke F, et al. SARS-CoV-2 in saliva, oropharyngeal and nasopharyngeal specimens. Dan Med J. 2021;68(5):A01210087. 33832565

[31] Cao Y, Li L, Feng Z, et al. Comparative genetic analysis of the novel coronavirus (2019-nCoV/SARS-CoV-2) receptor ACE2 in different populations. Cell Discov. 2020;6:11. 10.1038/s41421-020-0147-1 32133153 PMC7040011

[32] Masse S, Bonnet C, Vilcu AM, et al. Are posterior oropharyngeal saliva specimens an acceptable alternative to nasopharyngeal sampling for the monitoring of SARS-CoV-2 in primary-care settings? Viruses. 2021;13(5):761. 10.3390/v13050761 33926069 PMC8145717

[33] Erkhembayar R, Dickinson E, Badarch D, et al. Early policy actions and emergency response to the COVID-19 pandemic in Mongolia: Experiences and challenges. Lancet Glob Health. 2020;8(9):e1234–41. 10.1016/S2214-109X(20)30295-3 32711684 PMC7377809

